# STITCHER: Dynamic assembly of likely amyloid and prion β-structures from secondary structure predictions

**DOI:** 10.1002/prot.23203

**Published:** 2011-09

**Authors:** Allen W Bryan, Charles W O’Donnell, Matthew Menke, Lenore J Cowen, Susan Lindquist, Bonnie Berger

**Affiliations:** 1 Harvard/MIT Division of Health Science and Technology, Bioinformatics and Integrative GenomicsE25-519 Cambridge, Massachusetts 02139; 2 Whitehead Institute for Biomedical Research, Nine Cambridge CenterCambridge, Massachusetts 02142; 3 MIT Computer Science and Artificial Intelligence Laboratory, The Stata CenterCambridge, Massachusetts 02139; 4 Department of Computer Science, Tufts UniversityMedford, Massachusetts 02155; 5 Investigator, Department of Biology, MIT, Howard Hughes Medical Institute, Department of BiologyMIT, Cambridge MA 02139; 6 Department of Mathematics, Massachusetts Institute of Technology2-373, Cambridge, Massachusetts 02139 77 Massachusetts Ave., Cambridge, MA 02139

**Keywords:** structure prediction, β-sheets, energy model, stacking, entropy, dynamic programming

## Abstract

The supersecondary structure of amyloids and prions, proteins of intense clinical and biological interest, are difficult to determine by standard experimental or computational means. In addition, significant conformational heterogeneity is known or suspected to exist in many amyloid fibrils. Previous work has demonstrated that probability-based prediction of discrete β-strand pairs can offer insight into these structures. Here, we devise a system of energetic rules that can be used to dynamically assemble these discrete β-strand pairs into complete amyloid β-structures. The STITCHER algorithm progressively ‘stitches’ strand-pairs into full β-sheets based on a novel free-energy model, incorporating experimentally observed amino-acid side-chain stacking contributions, entropic estimates, and steric restrictions for amyloidal parallel β-sheet construction. A dynamic program computes the top 50 structures and returns both the highest scoring structure and a consensus structure taken by polling this list for common discrete elements. Putative structural heterogeneity can be inferred from sequence regions that compose poorly. Predictions show agreement with experimental models of Alzheimer’s amyloid beta peptide and the *Podospora anserina* Het-s prion. Predictions of the HET-s homolog HET-S also reflect experimental observations of poor amyloid formation. We put forward predicted structures for the yeast prion Sup35, suggesting N-terminal structural stability enabled by tyrosine ladders, and C-terminal heterogeneity. Predictions for the Rnq1 prion and alpha-synuclein are also given, identifying a similar mix of homogenous and heterogeneous secondary structure elements. STITCHER provides novel insight into the energetic basis of amyloid structure, provides accurate structure predictions, and can help guide future experimental studies.

Proteins 2012. © 2011 Wiley Periodicals, Inc.

## INTRODUCTION

Amyloid is a highly-ordered cross β-protein aggregate that can be achieved by a very broad set of proteins with widely divergent and unrelated amino acid sequences.^1^,^2^ Given the right conditions, a great many, perhaps most, proteins have the potential to form amyloids. The tendency towards amyloid appears to be because of intrinsic properties of the peptide backbone, a finding of great importance for understanding the evolution of protein folds. A much smaller fraction of proteins and protein fragments, assemble into amyloid under normal physiological conditions, and these are of great interest in diverse aspects of biology and medicine.^3^

Early amyloid research concentrated on amyloids associated with a wide variety of mammalian diseases, from systemic immunoglobulin amyloidoses to neurodegenerative diseases such as Alzheimer’s.^4^ Initial assumptions that accumulated amyloid always caused cellular and tissue toxicity, as is still believed to take place in peripheral amyloidoses,^5^ proved to be unfounded upon the discovery of a wider variety of amyloids. Amyloids are now known to play roles in bacterial biofilms,^6^ the production of melanin,^7^ the storage of hormones in secretory granules,^8^ and neuronal learning and memory.^9^ A set of self-templating fungal amyloids additionally give rise to epigenetic heritable traits. These bistable “prion” proteins can persist as soluble or amyloid species with different functional activities. The self-templating property causes cell-wide persistence of one or the other stable state, a status passed from generation to generation via cytoplasmic transfer of amyloid templates from mother to daughter cells.^10^,^11^ Increasingly, evidence suggests that the formation of amyloids may more commonly be a protective mechanism, which especially in the case of the neurodegenerative amyloidoses, acts as to sequester misfolded polypeptides that would otherwise dwell in more toxic, and more highly interactive, oligomeric species. Therefore, there is great interest in deciphering the structures that underlie amyloid states.

While the secondary structure of amyloid is known to be highly β-rich,^12^–^16^ experimental structural determination has proven highly difficult, with only extremely short segments crystallized^17^,^18^ and a very few successful solid-state nuclear magnetic resonance (ssNMR) studies.^19^–^23^ Because of the scarcity of direct evidence, the nature of amyloid and prion supersecondary structures, and their relation to sequence have been highly contentious topics.^17^,^24^, ^25^ The parallel β-helices form a fold widely cited as one-potential model for amyloid,^26^–^28^ while others favor a ‘superpleated β-sheet.’^29^–^31^ Complications include the morphological heterogeneity of amyloid structures suggested by EM imagery^27^,^32^ and the demonstration of prion ‘strains’ or ‘variants’ with differing growth and stability phenotypes.^33^–^35^ In the case of the yeast prion protein Sup35, such variants have been demonstrated to maintain specificity through serial passage^34^ and have been correlated with differences in conformation.^36^

The bistable nature of amyloid prions, as well as the observation of heterogeneity and ‘strains’ in amyloid and prion folding, undermines the canonical viewpoint of ‘one-protein, one-fold’ long held by theorists of protein folding. Instead, a murky view arises of a set of stable valleys in a field of conformational configurations, within which variations are permitted around common or similar folding patterns. Enzymologists have long studied the variations in globular protein conformations caused by ligand binding, catalytic activity, presence of ions or cofactors. Amyloids embody a similar but larger set of variations.

Bryan *et al*.^37^ and others have proposed that β-strand *pairs* form the core energetic subunits that make up amyloid structure, and a β-strand predictor was designed around this named BETASCAN. BETASCAN calculates likelihood scores for potential β-strands and strand-pairs based on correlations observed in parallel β-sheets. A key and novel feature of BETASCAN was a maxima-finding algorithm that searched the strands and pairs with the greatest local likelihood for all of the sequence’s potential β-structures. While sufficient to predict sequence regions with high potential for amyloidogenic β-structure, BETASCAN did not suggest the most likely overall β-sheet fold. For example, BETASCAN was unable to distinguish between the highly similar amyloidal HET-s allele and nonamyloidal HET-S allele in *Podospora anserine.*

The STITCHER method described in this article extends prediction of amyloid-like proteins by employing a combination of probabilistic prediction^38^ and free-energy^39^ methods for protein structural prediction. Since, few atomic-detail templates exist from known structures, the algorithm proceeds via a dynamic assembly of β-strands in agreement with the twists and turns necessary to form a β-helix or superpleated-sheet fold. This philosophy of establishing and then manipulating predefined structural components has been previously used successfully.^40^,^41^ The score of each successive β-strand addition is determined through a combination of novel free-energy model and BETASCAN-derived probabilities. The free-energy methods account for the enthalpy of created hydrogen bonds and the entropy of linkers, while the probabilities describing the likelihood of β-sheet formation account for the specific side-chain–side-chain interactions that confer structural stability. Of particular importance to our energetic model is the detection of stacking ladders, formed by the side-chain–side-chain stacking and bonding of glutamine, asparagine, tyrosine, and phenylalanine residues.^42^–^44^ To capture the observed structural heterogeneity of amyloids (e.g., the “strain” phenomenon), STITCHER calculates a list of the top-scoring *M* = 50 structures instead of just a single optimal. From this set of high-scoring candidates, a consensus structure is derived to represent the commonalities in specific strand-pairs among these 50 structures. Specifically, portions of the structure are considered more likely to form if they are seen in 80% or more of the top structures.

In our results, we show that the STITCHER method can be used to accurately reconstruct structure, as is given by the example of the well-studied Alzheimer’s amyloid beta-peptide and the *Podospora anserina* Het-s prion. STITCHER is also shown to be less prone to false positives than the prior BETASCAN program as it distinguishes the amyloid forming HET-s protein from its close, nonamyloidal homolog, HET-S. Novel structural predictions are then analyzed for the prion domain of the yeast protein Sup35 as well as the Rnq1 prion and alpha-synuclein. The STITCHER algorithm may be accessed at http://stitcher.csail.mit.edu.

## MATERIALS AND METHODS

## Algorithmic strategy

The greatest problem in protein structure prediction is the reduction of possible conformation patterns from a mind-boggling potential space to the few viable and stable conformations. In the present case, the restriction to parallel, all β-structures massively reduces the conformational space and simplifies the prediction problem. The maxima-finding algorithm of BETASCAN further reduced the space of solutions to only discrete secondary structural elements, identifying locally probable strands and strand-pairs. While this level of detail is useful in some cases, supersecondary, and tertiary structural information has been completely lost. The goal of STITCHER is to build upon the successful probability models of BETASCAN to then reconstruct supersecondary structures based on a minimal set of additional energetic features and constraints. While complex hydrophobic and kinetic interactions are known to affect the rate of *in vivo* supersecondary structure formation, STITCHER relies on this minimal set of energy parameters to make the simplest possible model of thermodynamically possible stable structures that could form. Specifically, STITCHER identifies the importance of stacking pairs and entropic penalties for β-strand linkers. Further, β-strands are assembled into full structures using parameters that restrict the length of linkers and the distance between paired strands, parameterized by an interpretation of tertiary β-helix or superpleated-sheet fold. A dynamic program is used to combine (“stitch”) BETASCAN strand-pairs using these energetic features and physical constraints, and outputs a list of the top 50 scoring assemblies. Dynamic programs utilize the fact that the calculation of scoring assemblies will reuse many smaller calculations. A piece of pseudocode may demonstrate how STITCHER uses the principle:

Calculate_beta_sheet_score (rung_number):If lookuptable (beta_sheet_score (rung_number – 1)) exists:All_but_last_rung_score = lookuptable (beta_sheet_score (rung_number – 1))ElseIf rung_number = 0:All but last_rung_score = Calculate_rung_score (first_ rung)Else:All_but_last_rung_score = Calculate_beta_sheet_score (rung number)End_ifLookuptable (beta_sheet_score (rung_number – 1)) = All_but_last_rung_scoreEnd ifCalculate rung_score (last_rung)Beta_sheet_score = rung_score + All_but_last_rung_scoreReturn Beta_sheet_scoreEnd Calculate_beta_sheet_score

In the pseudocode above, we show a simplified version of a portion of our algorithm calculating a score for a partial structure. First, the algorithm checks if a smaller piece of the structure—namely, all but the last rung—has already been scored. If so, it reuses that score without the need to repeat a calculation. If it is not scored, it calculates that smaller partial structure’s score and stores it. Crucially, for any structure larger than one rung, the algorithm recurses, storing the smaller structures’ scores along the way. It then scores the additional rung and stores that score as well. By using a recursive algorithm to make calculations only as needed and storing results for reuse, any partial structure need only be calculated once, saving greatly on calculation time and resources. When no more additional structure is available, the completed structure can then be considered for possible output depending on its score. Once a list of top candidate structures is assembled, we use polling to assess their agreement or disagreement for specific strand-pairs.

## Fold constraints and parameters

STITCHER constrains the assembly of strand-pairs to a limited space of amyloid-like parallel β-sheets. Following the conventions of previous authors^26^,^37^,^45^,^46^ and the evidence from known amyloid models,^20^–^22^ ^29^,^47^ we define an arrangement of *n* possible sheets, discretized by rungs. Each rung contains *n* strands, each contributing to a sheet. For a structure of *m* rungs there will be *mn* strands and *mn −* 1 *linkers* connecting strands to each other. Every rung is connected by strand-pairs of length *L*, identified by residues *i* and *j* stacked atop one another {*i*,*j* ≥ *i*, *L* > 1}. Therefore each structure contains (*m* − 1)*n* strand-pairs in each putative structure. A complete amyloid protofibril is modeled as many copies of such structures.

Bounds on the parameters *m* and *n* further reduce the number of choices to be made in selecting plausible structures. The two models of amyloids in the literature may be described by constraints on parameters. The β-helix fold requires *m* ≥ 1, *n* ≥ 2^19^,^21^,^48^; nearly all observed cases in nature, including solved structures of amyloids, are either *n* = 2 or *n* = 3. A simple model of a superpleated β-sheet is formed by parameters *m* = 1, *n* ≥ 2. In this model every copy of the amyloid protein forms a single rung of *n* β-strands. In the case of *m =* 1, therefore, every strand-pair consists of two identical copies of a strand, and *i* = *j* for all strand-pairs. Therefore, two possible sets of parameters were considered for analysis: *n* = {1,2,3}, *m* ≥ 1 and *m* = 1, *n* ≥ 1, *i* = *j*.

Finally, we restrict the space of potential strand-pair combinations such that *m* > 1 amyloids must loop back to a location near their starting point at the end of each rung. Therefore, the distance from the end of the first strand of any strand-pair to the start of the second must be longer than the strand itself: (*j* − *i*) >2*L*. Likewise, strand-strand linkers can be no shorter than three residues long—the tightest turn observed in crystal and NMR structures.

## Free-energy scoring function

Amyloid structures are scored using a formula that includes the BETASCAN scores of their strand-pairs, as well as bonuses reflecting the stabilizing influence of nonbackbone hydrogen bonding, and the entropic cost of restricting backbone movement into the loops of strands. The weight of each component in the scoring function was determined by summing estimates of their free-energy contributions to stability. Following the pattern of Zhang *et al.*,^49^ the Gibbs free energy delta G of the change from unfolded to folded state is


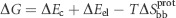


where Δ*E*_c_ is the contact enthalpy of placing residues together, Δ*E*_el_ is the electrostatic energy associated with ionic interactions, and 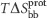 is the entropy of folding. Amyloids are typically sparse in charged amino acids, and the stronger partial dipoles associated with other amino acids are usually incorporated into hydrogen bonding. In this analysis, the electrostatic interaction is therefore only considered with reference to hydrogen bonds, and Δ*E*_el_ is set to zero. Contact energies can be further decomposed into energies contributed by backbone–backbone, backbone–side-chain, and side-chain–side-chain interactions:


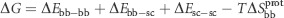


## Role of BETASCAN scores

We model amyloid peptide backbones to contain only β-strands, with a linear arrangement constrained by the hydrogen bonds to other strands and linkers, which are only constrained by the strands at their beginning and end. There is one hydrogen bond per residue in the length of each strand-pair, and an equivalent entropy loss for the constraint it imposes on backbone flexibility. Therefore, we separate the entropy into linker and strand terms, and rearrange to express them with reference to length:


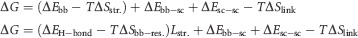


Side-chain/backbone interactions are a primary determinant of β-sheet propensities, both through van der Waals interactions^50^ and entropy of solvation.^51^ These factors explain much of the known relative affinities of amino acids,^52^ although these propensities must be interpreted in context.^53^ The BETASCAN algorithm uses these propensities to estimate relative probabilities of formation of a strand-pair, normalized by length to allow comparison of strands with different lengths. This log-odds estimate of the relative probability of a β-strand conformation is applied to Δ*G*_β form_. The energies from the direct hydrogen bonds made by all residues in the strand may be combined with the side-chain effects and estimated by the entire strand score:


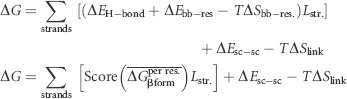


## Side-chain stability bonuses

Side-chain/side-chain energies include hydrogen bonding in the case of asparagine and glutamine stacking,^42^ pi-bond orbital stacking in the case of tyrosine and phenylalanine,^54^ and van der Waals interactions between side-chains. The first two contribute bonus stability beyond that calculated by BETASCAN. The last has been shown to be very small (less than 0.20 kcal/mol^49^) and can be disregarded. We therefore set:


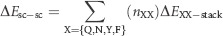


## Entropic penalties

Finally, we consider the entropy of the linkers, defined as free loops of peptide between β-strands. We note, but do not include here, the difficult problem posed by the paradoxical contributions of polyglutamines^55^ to the entropy of β-structures such as the huntingtin fibril. The problem of calculating the linker entropy is otherwise a subset of the general problem of polymer condensation entropy^56^ and bears remarkable similarity to that of disulfide bond entropy.^57^ The entropy may be calculated as


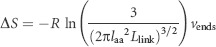


where *R* is the gas constant, *l*_aa_ the length from α-carbon to α-carbon, 3.8 Å, *L*_link_ is the number of residues in the linker, and *v*_ends_ is the volume the ends of the linker may occupy. A hydrogen bond is approximately the same length as the distance between sulfide groups in a disulfide bond, namely 4.8 Å. The entropy calculation, using these values, may be simplified to


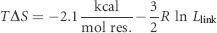


for *T* ≍ 300 K. Because we are comparing structures known to have linkers, we disregard the constant term and make an estimate yielding the relative entropy of a linker,


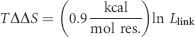


This formula is used in two different ways: to calculate the entropic penalty of adding β-strands extending a sheet and to calculate the entropic penalties accrued from combining multiple sheets into the fiber. The difference is in the value of *L*_link_. For the former, we assess the entropy of forming a loop from a free polypeptide-chain without regard to strand-pairs (as the strand-pairs cannot form until the chain is in proximity to itself). In this case 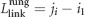 is the difference between the N-termini of the two strands. For linkers between two separate β-sheets *f* and *g*, the length of the linker is the number of residues between strand-pairs, counting both the upper- and lower-chains of the pair:


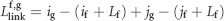


The form of the scoring function for STITCHER may now be fully described as


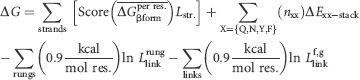


## Energy weights

To calculate this function, we must estimate 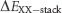. Experimental data^49^–^51^,^53^ suggests the free-energy of β-strand formation per residue to be ∼ 1 kcal/mol res, a combination of the enthalpy of the hydrogen bond and the entropy of solvation as influenced by side-chains. This is a somewhat rough estimate because of context-dependency.^53^ For the bonuses and penalties, we assess the contribution of additional hydrogen bonds to the free-energy. The free-energy of the hydrogen bond is again offset to some degree by solvation, though not as strongly as for the backbone. The rough bonus weights 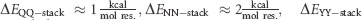

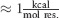
were used for this study. The extra weighting of *NN* over *QQ* is justified in two ways. First, asparagine (*N*) has a shorter distance from backbone to amide than does glutamine (*Q*). Additionally, experimental data^43^ suggests that in at least one prion, replacing all glutamines with asparagines provides better stability of the prion-fold as compared to replacing all asparagines with glutamines. These estimated energy weightings may become more accurate as calorimetry of side-chain–side-chain interactions becomes available.

## Evaluation of score

The STITCHER algorithm uses a dynamic programming algorithm to evaluate estimated Δ*G* for the combinations of strand-pairs that can be combined into templates matching the parameters described earlier. To do so, the calculation of Δ*G* is subdivided into calculation by rungs. The total free-energy change can then be calculated by summing the stability contribution of any rung *r* containing strands {*r*_1_…*r*_*n*_} and linkers 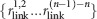, with a linker 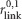 to the previous rung, as


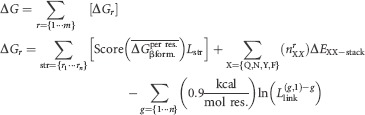


If the stacking bonuses, a directly sequence-dependent calculation, are considered apart from the strand and linker scores, which are only indirectly sequence-dependent, then the rung calculation can be partially separated into strand calculations:


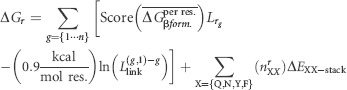


By calculating the free-energy scores of strand-pairs and linkers as subproblems of rung scoring, and rung scores as subproblems of structure assembly, the dynamic programming method can be used to iteratively calculate the *M* structures with the highest score by tracing back through internally consistent partial structures that do not violate the defined fold constraints.

## Evaluating consensus outputs

The composition of the *M* highest structures (with default *M* of 50) is assessed by scanning over all structures for included strand-pairs by the locations (*i*, *j*) of their termini. If the number of strand-pairs in the *M* highest structures with N-termini of (*i* ± 2, *j* ± 2) total more than 80% of *M*, the location is noted as a consensus structure element, and the ranges of *i*, *j*, *L* and strand-pair score over the strand-pairs in the (*i* ± 2, *j* ± 2) region are output. For display purposes, the strand-pairs with matching lower and upper strands are aligned vertically to reconstruct predicted β-sheets. The output of STITCHER includes the set of *M* predicted structures, a diagram of local structure space, and the top scoring consensus structure.

## RESULTS AND DISCUSSION

### Amyloid-beta

**Figure 1:**
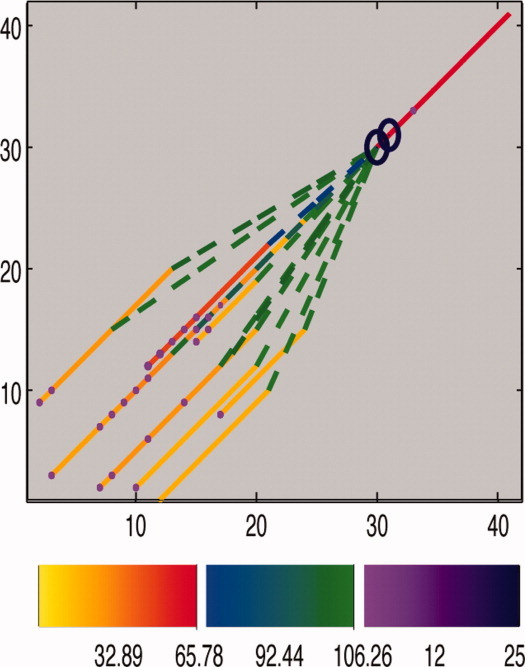
STITCHER results for Aβ (amyloid beta) (*m* = 1). At left, a contact map of the 50 highest-scoring folds. The horizontal and vertical axes indicate, respectively, the residue numbers (counted from the N-terminus) of the lower and upper strands in each strand-pair of the structures. Starting locations of strand-pairs are indicated by circles, with size of circle (small to large) and color of circle (magenta to black, right-hand color spectrum) indicating the strength of the vote of the top 50 structures for that strand-pair. The strand-pairs are drawn along their length in shades of orange, with stability increasing from yellow to red (left-hand color spectrum). Fold structures are indicated by the dotted lines connecting strand-pairs into rungs and sheets. Structure scores are indicated by shades with stability increasing from blue to green (center color spectrum). At right, the highest-scoring fold. Each strand-pair is denoted by its score (Sc) and its length (L). To the extent possible, rung-pairs proceed from left to right and sheets from top to bottom. Numbers to the left of the strands indicate the number of the residue immediately preceding the N-terminus of the strand. Slanted lines indicate the first residue of the strand, arrowheads the last residue of the strand, and connecting line(s) indicate the possible residue-pairing(s) of the first residues of the strands.

STITCHER was tested on amyloid beta, an amyloid with two experimental NMR models,^20^,^47^ allowing both superpleated sheet (*m* = 1, *i* = *j*) and β-helix (*m* > 1) structures (see symbols used for definitions of all variables). The results are shown in Figure 1. In the case of amyloid beta, the highest-scoring structures all incorporate at least one *i* = *j* strand-pair (the 10-residue β-strand from isoleucine 31 to valine 40 inclusive). Several of the highest-scoring structures include strands analogs to those in solved NMR structures, including strands beginning at tyrosine 10 (corresponding to^47^) and at leucine 17 (corresponding to 20). However, the highest-scoring structures include a first strand with one residue shifted from a perfect in-register parallel alignment, in the region between histidine 13 and alanine 21 (sequence HHQKLVFFA). This region is known to exhibit structural variability, especially at differing pH.^58^

### Het-s/S

A key goal in amyloid folding studies is to distinguish amyloidogenic from nonamyloidogenic sequences. STITCHER was run on the small-s and big-S variants of the Het-s mating compatibility factor to test its ability to make this classification. The small-s allele of this protein is known to form an amyloidal prion.^21^,^22^ In contrast, the big-S allele does not form an amyloid structure.

**Figure 2:**
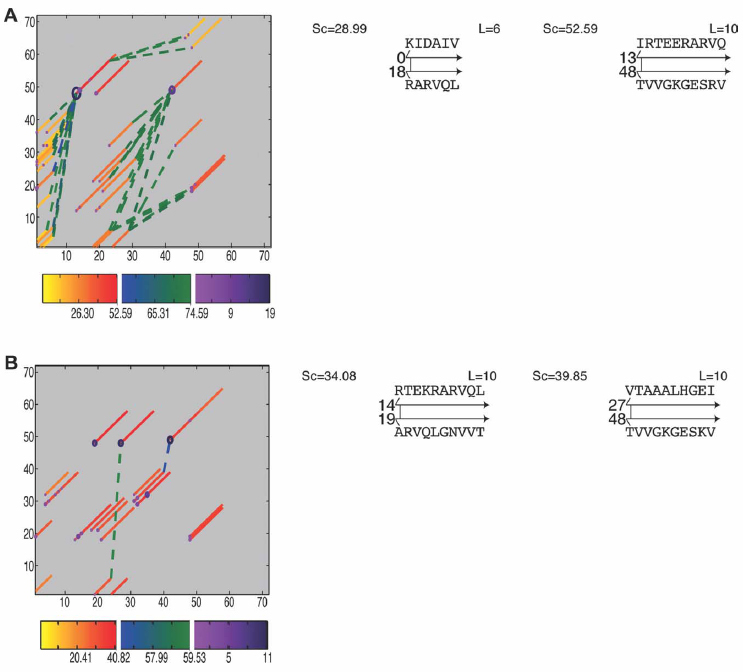
STITCHER results (*m* = 2) for the two alleles of the *Podospora anserina* mating compatibility protein: (A) Het-s and (B) Het-S. Contact maps, at left, and top-scoring structures, at right, are as described in the caption to Figure 1.

The results for Het-s and Het-S are displayed as Figure 2(A,B), respectively. Immediately, evident is the greater “connectivity” of Het-s predictions (i.e., the high number of valid sequential orderings of β-strand pairs). On the other hand, very few of the predicted Het-S strand-pairs are able to form multiple-sheet structures. Furthermore, the single HET-s strand-pair most often seen in the list of 50 top structures (identified by N-terminal residue-pair isoleucine 14–threonine 49) overlap with the conformation of strands 1b, 2a, 3b, and 4a in the most recently solved NMR structure,^22^ although off by one in pairing registry.

### Sup35p: case study in three species

**Figure 3:**
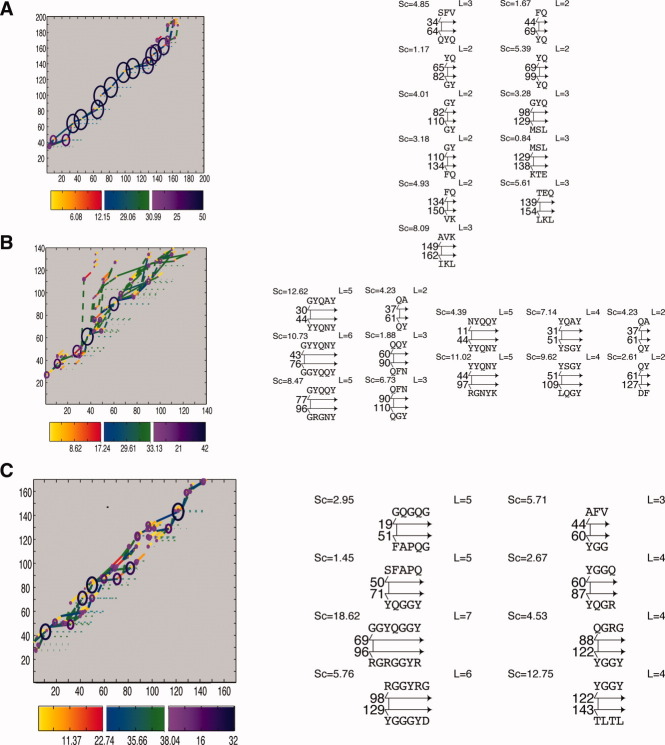
STITCHER results for Sup35p (*m* = 3) in three species. (A) *C. albicans*, (B) *S. cerevisiae*, and (C) *Y. lipolytica*. At left, a contact map of the 50 highest-scoring folds. At right, the top-scoring structure. For *S. cerevisiae*, the highest-scoring structures for *n* = 2 and *n* = 3 are presented. For description of colors, numbers, and lines, see the caption to Figure 1.

We used STITCHER to computationally investigate the amyloidogenic impact of minor sequence differences in homologs proteins. Three homologs of the yeast prion Sup35p were chosen, taken from *Candida albicans, Saccharomyces cerevisiae,* and *Yarrowia lipolytica*. The outputs of these predictions are shown in Figure 3.

The top 50 structural predictions for the *C. albicans* sequence suggest only a single possible folding route [Fig 3(A)], as all top structures contain recurrent β-strand pairs. The consensus structure thus stretches nearly the entire sequence. Further, the probabilistic signal from each structural strand-pair is fairly weak, with most strands consisting of only three or fewer residues. It appears that most of the amyloidogenic potential detected in this sequence comes from its long polyglutamine stretches. STITCHER accounts for these via loop-based stacking bonuses that stabilize β-sheet structure. Specifically, tyrosine and phenylalanine ladders serve to align the structure, in agreement with some mutagenic experiments.^42^

The top 50 structural predictions for Sup35p *S. cerevisiae* [Fig 3(B)] and *Y. lipolytica* [Fig 3(C)] are more heterogeneous. Much of this heterogeneity is due to the recurrent repeats in the Sup35p sequence. For instance, the strand beginning at glycine 44 can favorably be paired with the repeats at glycines 68, 77, and 97. Three pairs in the *S. cerevisiae* structure, identified by their N-terminal residues as asparagine 12–glutamine 38, tyrosine 29–tyrosine 49, and glutamine 38–glutamine 62, are highly recurrent. Interestingly, these pairs correspond to the “head” area identified in previous experimental studies,^36^ where mutations are known to have large effects on amyloidogenic potential.^59^ In contrast, predictions for the region beyond residue 91 shows increased variance. This “tail” region also exhibits experimental variability in different protein “strains.”^36^ The *Y. lipolytica* structure [Fig 3(C)] shows similar traits, but with fewer recurrent β-structures most likely as a result of the irregularity in sequence repeats.

### The effects of ladders and residue-repeats in Alpha-synuclein and Rnq1p

**Figure 4:**
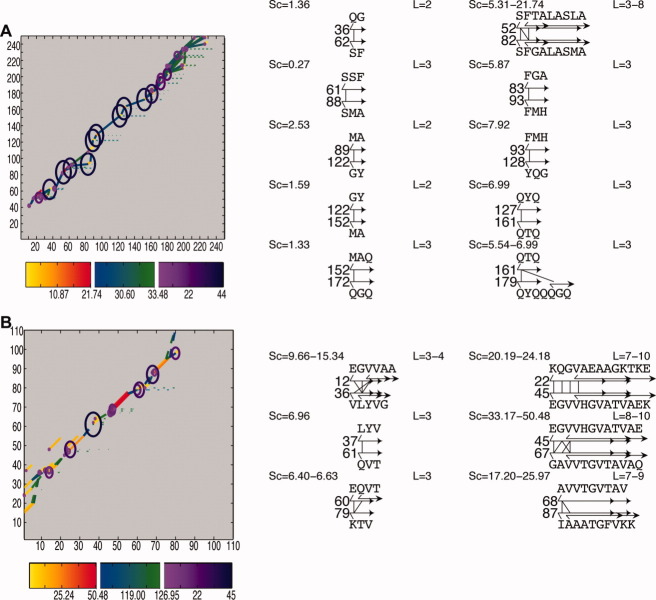
STITCHER results for (A) *S. cerevisiae* Rnq1p (*m =* 2) and (B) α-synuclein (*m* = 2). At left, a contact map of the 50 highest-scoring folds. At right, a consensus structure assembled from all clusters of strand-pairs 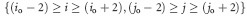 found in > 80% of the top 50 highest-scoring folds. The range of strand positions and lengths is indicated by the shortest and longest possible strand arrows, drawn at the most N- and C-terminal possible locations. Connecting lines indicate the possible pairings of the first residues of the strands making up the strand-pair. The range of lengths and scores for a set of possible strand-pairs is indicated at L and Sc, respectively, for each set. The numbers to the left of the strands indicate the residue immediately before the leftmost possible residue in each strand (i.e., the residue before the first written above and below the strand arrows). For description of colors and numbers, see the caption to Figure 1.

To further study the effect of residue side-chain stacking, STITCHER was also used predict the structures of two other important amyloidogenic proteins: human alpha-synuclein and the *S. cerevisiae* prion Rnq1p (Fig 4). Although, a putative structural model has been published for alpha-synuclein,^60^ no structure has been proposed for Rnq1p. However, it has been shown that Rnq1p may facilitate templating in Sup35p fibers.^61^,^62^

The top 50 structure predictions for Rnq1p [Fig 4(A)] follow a similar pattern as in *C. albicans* Sup35p, identifying many recurrent β-structures. Although, nonspecific loop-based side-chain ladders of glutamines contribute the most to the energetic score of these structures, shorter strands including glutamine, phenylalanine, tyrosine, and asparagine ladders also contribute to the identification of specific β-strand pairs. β-structure content is highest in the region of residues 53–175, albeit with large exterior loops exposing a high number of glutamine and asparagine residues. This suggests that polyasparagine and polyglutamine stretches limit highly specific β-strand pairing.

β-helical STITCHER predictions for alpha-synuclein [Fig 4(B)] output a strong signal for recurrent strand-pairs in the region roughly between residues 20 and 80. The highest-scoring structures are composed of two β-sheets; the first with strands of length 3 and the second with strands of lengths 7–10. Interestingly, some strand pairing regions exhibit alternate registries in nearly the same locale and with nearly equal scores. As with amyloid beta, this predicted variability is associated with the existence of repeated residues found near the edge of the strands (e.g., valines 15, 16, alanines 17–19; alanines 29, 30; valines 48, 49, glycines 67, 68, valines 70, 71, alanines 88–91, and lysines 96, 97).

## CONCLUSION

STITCHER introduces a novel energetic scoring model for amyloid fibrils and an efficient algorithm for dynamically assembling discrete β-strand pairs into complete amyloid structures. The system of physical constraints used to “stitch” β-strands into a complete structure offers an accurate generalization of successful template-based methods such as BETAWRAP and its successors,^44^,^51^,^52^ which only conformed to rigid templates. In addition, STITCHER takes into account the unavoidable uncertainty in free-energy parameters and the potential heterogeneity of amyloid folds by computing multiple solutions instead a single optimal. Although, the highest-scoring fold frequently offers a good solution, the best results are achieved by assembling the top solution’s most frequently occurring strand-pairs into consensus structures. It should be noted that the particular fold taken by an amyloid is sensitive to environmental conditions, including pH, concentration of protein, and presence of chaperone proteins. Thus, the structure with the highest STITCHER score may not be that taken by the protein under experimental conditions.

The results for alpha-synuclein, Rnq1p, and to some extent the various Sup35p proteins highlight the role of single-residue repeats, motif repeats, and sidechain stacking ladders in amyloid structure. Single-residue repeats may contribute to structural stability through stacking ladders, especially in the cases of polyglutamine and polyasparagine. However, this stability increase comes at the expense of β-strand pairing specificity, as the importance of aligning any particular pair of residues is reduced. A similar but diminished effect is seen in repeats composed of multiple-residue motifs, such as in *S. cerevisiae* Sup35p. This suggests that the β-strand pairing specificity for repeat-heavy amyloidogenic proteins may be conveyed through two other features of a structure: short intervening linker loops, and the formation of strand-pairs with more rare stacking ladders such as histidine and phenylalanine.

In the cases of known amyloid structures amyloid beta and HET-s, STITCHER is able to predict the core regions of β-structure observed experimentally. Conversely, the predicted results for Het-S agree with the protein’s observed nonamyloidogenic nature, despite a high β-propensity sequence that is nearly the same as the amyloidogenic HET-s prion. While a more robust analysis and verification would require additional amyloid structure determination, the STITCHER methodology appears to be a valuable addition to the growing number of amyloid detection algorithms. Further, as new experimental data provides better insight into the nature of β-strand energetics, and new amyloid structures arise, the STITCHER algorithm could be readily extended. While some interpretation and experimental verification is necessary for a complete understanding of amyloid folding, the identification of the range of most likely folds should greatly enhance the further computational and experimental investigation of amyloid and prion proteins.
